# *In Vivo* Hip Joint Loading during Post-Operative Physiotherapeutic Exercises

**DOI:** 10.1371/journal.pone.0077807

**Published:** 2013-10-29

**Authors:** Verena Schwachmeyer, Philipp Damm, Alwina Bender, Jörn Dymke, Friedmar Graichen, Georg Bergmann

**Affiliations:** Julius Wolff Institute, Charité – Universitätsmedizin Berlin, Berlin, Germany; West Virginia University School of Medicine, United States of America

## Abstract

**Introduction:**

After hip surgery, it is the orthopedist’s decision to allow full weight bearing to prevent complications or to prescribe partial weight bearing for bone ingrowth or fracture consolidation. While most loading conditions in the hip joint during activities of daily living are known, it remains unclear how demanding physiotherapeutic exercises are. Recommendations for clinical rehabilitation have been established, but these guidelines vary and have not been scientifically confirmed. The aim of this study was to provide a basis for practical recommendations by determining the hip joint contact forces and moments that act during physiotherapeutic activities.

**Methods:**

Joint contact loads were telemetrically measured in 6 patients using instrumented hip endoprostheses. The resultant hip contact force, the torque around the implant stem, and the bending moment in the neck were determined for 13 common physiotherapeutic exercises, classified as weight bearing, isometric, long lever arm, or dynamic exercises, and compared to the loads during walking.

**Results:**

With peak values up to 441%BW, weight bearing exercises caused the highest forces among all exercises; in some patients they exceeded those during walking. During voluntary isometric contractions, the peak loads ranged widely and potentially reached high levels, depending on the intensity of the contraction. Long lever arms and dynamic exercises caused loads that were distributed around 50% of those during walking.

**Conclusion:**

Weight bearing exercises should be avoided or handled cautiously within the early post-operative period. The hip joint loads during isometric exercises depend strongly on the contraction intensity. Nonetheless, most physiotherapeutic exercises seem to be non-hazardous when considering the load magnitudes, even though the loads were much higher than expected. When deciding between partial and full weight bearing, physicians should consider the loads relative to those caused by activities of daily living.

## Introduction

After hip surgery, such as total hip arthroplasty (THA), osteotomies or osteosynthesis of proximal femoral fractures, physiotherapy and mobilization usually begin on the first post-operative day. Early mobilization leads to faster recovery and reduces complications due to bed rest, such as thrombosis or pneumonia [1], [2]. Concurrently, many elderly patients are unable to walk with partial weight bearing due to insufficient arm strength or poor body control [3], [4]; therefore, many surgeons allow early full weight bearing.

The question has been addressed if immediate full weight bearing is detrimental for bone ingrowth in THA surfaces. When uncemented implant stems lack primary stability, micromotions at the bone-stem-interface may occur with high loads [5] and impair long-term fixation. Studies demonstrated that bone ingrowth into porous surfaces decreases with increasing micromotion: the larger the motion between the bone and the implant, the more the implant fixation is dominated by fibrous tissues instead of cancellous bone [6], [7]. As a result, on one hand, a lack of primary stability requires partial weight bearing for up to 12 weeks to ensure proper bone ingrowth. On the other hand, implant design, coating materials and implantation techniques have substantially improved over the last decades, increasing the primary stability of uncemented stems [8]–[11], thus indicating that partial weight bearing is not essential for bone ingrowth. Due to the controversial arguments, there is no consensus among orthopedic surgeons whether to allow early full weight bearing, and recommendations vary in clinical practice from immediate unrestricted weight bearing to partial or even toe-touch weight bearing for several weeks [4], [12]–[14].

For osteotomies or surgically stabilized femoral neck fractures, primary stability of the osteosynthesis is decisive for fracture consolidation. Depending on the location and complexity of the fracture, shear and bending forces or moments may delay or even hinder bone union [15], [16]. For inter- and pertrochanteric femoral fractures, failure rates of 10 and 40% have been reported [17]. The aim of any surgical intervention is therefore to provide a stable fixation to allow full weight bearing during activities of daily living. In some cases, this cannot be achieved or the weight bearing capacity of the fixation is questionable.

However, avoiding high loads at the fracture site or bone-stem-interface throughout the first post-operative weeks appears to be beneficial for optimal bone healing. A justified classification for ‘high’ or ‘low’ load levels depends on the investigated implant, the fracture situation, the disease, and several other factors; therefore it cannot be generalized. However, it is impossible to provide *general* exact thresholds for forces or moments which are detrimental for osteoarthritis or the outcome of surgical interventions. Most studies that tested the primary stability of implants used force magnitudes based on Bergmann’s findings [18], [19]. As the primary stability depends on several factors, the tolerable load levels would have to be individually defined. Here, high loads are considered those that overload the surrounding musculoskeletal structures and thereby result in possible damage. Particularly during the most frequent activities of daily living (ADL), which include walking, standing and going up or down stairs, cyclic or permanent high loads may be detrimental. Previous *in vivo* investigations have measured peak hip contact forces of approximately 250% of the patient’s bodyweight (BW) during level walking and torsional moments of 1.6%BWm around the implant axis [18]. During stumbling, forces of nearly 900%BW were measured [20]. Whereas the loading conditions during most ADL are known, it remains unclear how demanding physiotherapeutic exercises are. Only one study investigated the hip contact forces during physiotherapy [21], which were measured telemetrically using an instrumented joint implant. The data were collected in only one patient and the loading situations were not precisely defined.

The aim of this study was to augment this knowledge by systematically measuring the hip contact loads during physiotherapeutic exercises *in vivo* in a cohort of 6 patients. This study focuses on the resultant joint contact force, the bending moments in the femoral neck and the torque around the implant stem axis, as these are the three most important mechanical factors for THA, osteotomies, femoral neck fractures, and coxarthrosis [19].

## Materials and Methods

### Subjects

Six patients (5 male, 1 female, mean age 58±7 years, body mass 86±6 kg, height 174±5 cm) with instrumented hip endoprostheses were investigated. In every patient, advanced hip osteoarthritis was confirmed and indications for total hip replacement were given. The study was approved by the Charité Ethics committee under the registry number EA2/057/09 and registered with the ‘German Clinical Trials Register’ (DRKS00000563). All patients gave written informed consent prior to participating in this study.

### Physiotherapeutic Exercises

Prior to the evaluations conducted for this study, we repeatedly measured peak forces during the exercises within the first post-operative year to investigate possible changes over time. Such changes were not observed, as shown by sample measurements provided in the data base www.OrthoLoad.com. Therefore, we present data from time points when the patients were able to perform the exercises without pain. Subject #4 reported pain in the contralateral hip during exercise #4; it was therefore excluded from the analysis for this patient. The finally selected and evaluated measurements were taken between the 5^th^ and 12^th^ post-operative month, except from exercise #11 with data taken from the 4^th^ postoperative week.

All patients followed an investigation protocol that included 13 common physiotherapeutic exercises (Table 1) which were performed on a therapy table. The selection included weight bearing exercises with closed kinetic chains (exercises #1–#4), isometric exercises in which the patient was instructed to actively contract his/her muscles (#5–#7), exercises with the force acting at a long lever arm (#8, #9), and simple dynamic exercises in the supine position (#10–#13). Instructions were given by an experienced physiotherapist who also ensured that all exercises were performed correctly without compensational movements that could influence the acting loads.

**Table 1:** Description of 13 physiotherapeutic exercises.

Number	Exercise	Description
**1**	Lifting pelvis (Bridging) maximally	Supine position: knees flexed, feet standing on therapy table, arms at rest on table surface beside trunk. Pelvis lifted maximally.
**2**	Lifting pelvis (Bridging) slightly	Supine position: knees flexed, feet standing on therapy table, arms at rest on table surface beside trunk. Pelvis lifted slightly.
**3**	Lifting pelvis (Bridging) one legged standing on ipsilateral leg	Supine position: knees flexed, feet standing on therapy table, arms rest on table surface beside trunk. Pelvis and the contralateral leg lifted.
**4**	Lifting pelvis (Bridging) one legged standing on contralateral leg	Supine position: knees flexed, feet standing on therapy table, arms rest on table surface beside trunk. Pelvis and the ipsilateral leg lifted.
**5**	Isometric contraction; flexed knees	Supine position: feet standing on surface. Dorsiflexion, heels push into surface, gluteus maximus contracted, pelvis tilted posteriorly.
**6**	Isometric contraction; straight knees	Supine position: dorsiflexion, knee hollows push onto the therapy table surface (active knee extension), gluteus maximus contracted.
**7**	Isometric hip abduction	Supine position: Straight leg, patient pushes isometrically against external force transducer as strong as possible without pain.
**8**	Hip abduction with straight knee	Lateral position: hip abduction with dorsiflexion, extended knee, slight hip internal rotation. Strict supervision to prevent abdominal musculature, hip flexors or quadratus lumborum muscle being used for compensating possible weakness of abductor muscles.
**9**	Hip flexion with straight knee	Supine position: straight leg, hip flexed to about 30° and held for 4 seconds.
**10**	Dynamic hip abduction	Supine position: leg abducted and adducted dynamically back to original position while heel drags over surface, limb is only slightly lifted.
**11**	Hip and knee flexion/extension; heel on bench	Supine position: hip and knee flexed, heel drags over surface, limb is not lifted entirely.
**12**	Pelvis tilt	Supine position: feet standing on surface, pelvis tilted anteriorly (Hyperlordosis).
**13**	Pelvis tilt	Supine position: feet standing on surface, pelvis tilted posteriorly (Hypolordosis).

Every patient repeated the physiotherapeutic exercises 8 times. The first and last repetitions were excluded from the evaluation; the first one because verbal instructions slowed the movement down and the last one because the patients tended to perform it faster. As a result, 6 repetitions were included in the analysis. Each subject additionally walked 5 times along a 10 m walkway on level ground and the data from 10 walking cycles were analyzed.

### Instrumented Implants

The *in vivo* forces and moments were measured using instrumented hip implants with an inductive power supply and telemetric data transmission. Clinically proven, standard implants (type CTW, Merete Medical GmbH, Berlin, Germany) with a titanium stem and 32-mm Al_2_O_3_ ceramic head were equipped with 6 internal strain gauges to measure the deformations in the implant neck. By applying complex calibration loads and procedures, 3 force and 3 moment components were calculated from the deformations with an accuracy of 1–2%. All forces and moments were normalized to the patient’s body weight and are reported as %BW and %BM*m, respectively. The data from implants on the left side were mirrored to the right side. Further details have been described previously [22].

### Coordinate Systems

**Figure 1:**
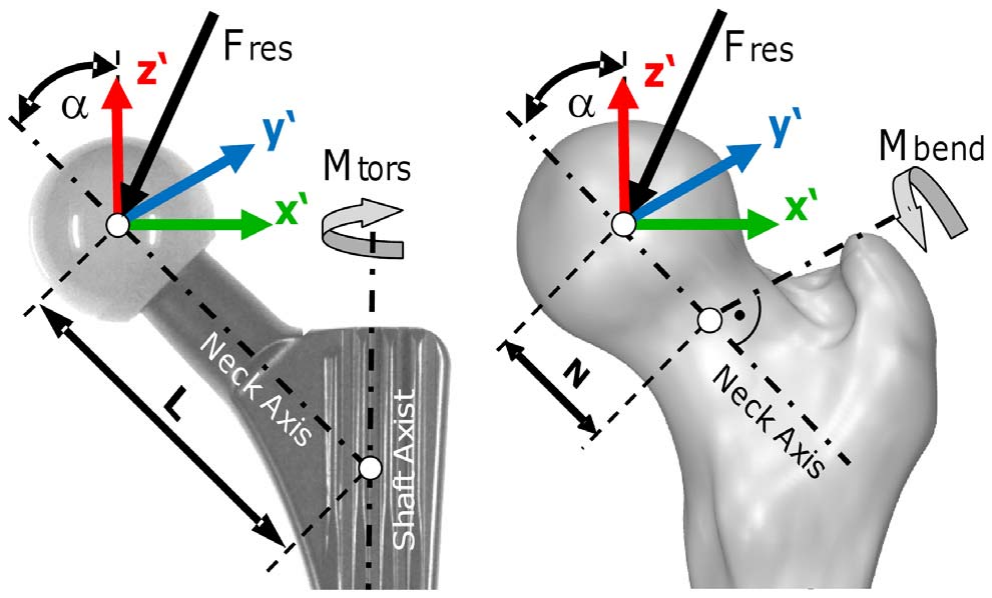
Resultant force, torsional moment around the implant stem and bending moment in the femoral neck. View from posterior. The torsional moment M*_tors_* rotates the implant backwards around its stem axis. The bending moment M*_bend_* acts in the middle of the femoral neck. α = CCD angle.

The forces and moments were measured in the implant system x′, y′, z′, centered in the middle of the head (Figure 1). The plane x′/z′ is formed by the implant neck and the long axis of the femur. The force component F*_x_*_′_ acts laterally, F*_y_*_′_ anteriorly, and –F*_z_*_′_ distally along the femur axis. F*_res_* is the resultant force, consisting of all 3 components. The moment components M*_x_*_′_, M*_y_*_′_, and M*_z_*_′_ turn right around the x′, y′, and z′ axes.

### Evaluated Loads

Three types of loads were evaluated (Figure 1):

1. The resultant contact force F*_res_* consists of its 3 components:
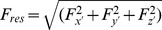
(1)

1. The torsional moment M*_tors_* acts around the implant’s stem axis and rotates the implant inwards when positive. With α = 45° being the angle between the implant’s stem and neck axes, and L being the length of the implant neck, given by the distance between the center of the implant head and the point of intersection of the neck axis and the implant shaft axis, M*_tors_* is calculated by the following equation:
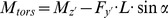
(2)

1. The bending moment M*_bend_* acts in the middle of the femoral neck, perpendicular to the neck axis:
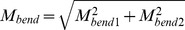
(3)with 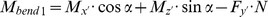


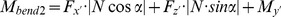

N is the distance between the head center and the middle of the femoral neck and equals L/2. The direction of M*_bend_* is not reported here.

### Analysis of Time-load Patterns

The time-load patterns of F*_res_,* M*_tors_* and M*_bend_* were averaged throughout the entire exercise. A dynamic time warping algorithm [23] was used to deform the time scales of the 6 repetitions, so that the summed squared errors between the 6 F*_res_* patterns became a minimum. These time-deformed forces were then averaged arithmetically and delivered the ‘patient-specific’ time course of F*_res_* for this exercise. The ‘patient-specific’ curves from all 6 patients were averaged again, using the same algorithms, which finally delivered the ‘activity-specific’ time pattern of F*_res_*. The time deformations obtained when averaging F*_res_* were then applied to the M*_tors_* and M*_bend_* patterns before averaging their time patterns, too.

### Analysis of Load Maxima

**Figure 2:**
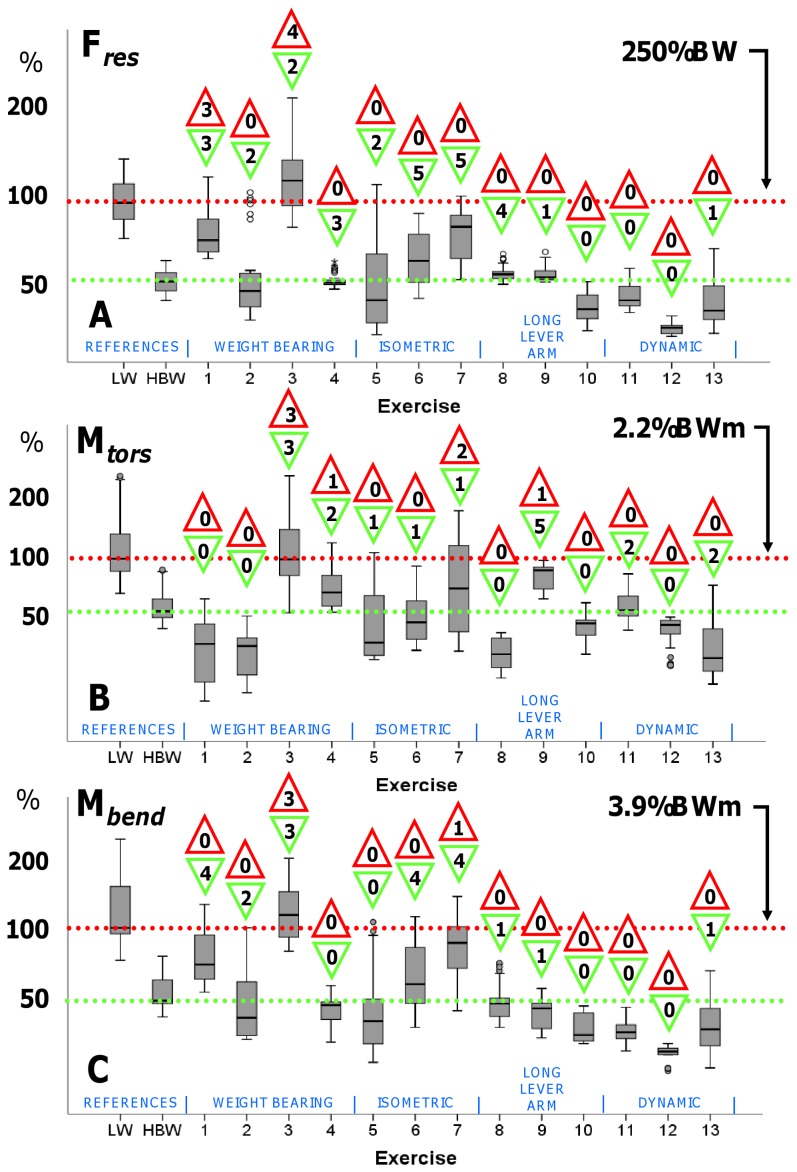
Median peak loads. Median Peak values of Fres (A), Mtors (B), and Mbend(C) and their ranges for the reference activities Level Walking (LW) with full ( = 100%) and half ( = 50%) weight bearing as well as the 13 physiotherapeutic exercises. Horizontal lines mark the activity-specific median peak value from walking. See Table 1 for exercise numbers descriptions. Walking at 100% level (with full weight bearing) and 50% level serve as reference for comparison. The numbers in the upper triangles indicate the number of patients having high loads, the number in the triangles below indicate the number of patient, in which medium loads were found.

The absolute maxima of F*_res_*, M*_tors_* and M*_bend_*, acting within each single trial, were determined for the 6 repetitions of each of the 6 patients, resulting in 36 peak values for every exercise (30 for #4). An exploratory data analysis was performed on the 3 load maxima and depicted as box plots in Figure 2. The same procedure was performed for the 10 walking cycles of each patient.

For defining high and low loads and enabling an interpretation of the measured data, we used the peak load values during walking as references. The median peaks of F*_res_*, M*_tors_* and M*_bend_* during walking with full weight bearing were set to 100% and exercise loads higher than these limits were classified to be ‘high’. Loads were named ‘medium’ if their peak values lay between 50% and 100% of these limits, and ‘low’ if they were lower than 50%. These classifications are based on clinical considerations: If a surgeon allows the patient to walk without support, physiotherapeutic exercised causing medium and even high loads should also be tolerated. If only walking with half body weight is permitted, physiotherapeutic exercises which cause medium or even high loads should consequently be avoided.

Separately for each exercise, the individual median peak values of F*_res_*, M*_tors_* and M*_bend_* were compared to the 100% and 50% levels of the *same* subject, using a Student’s-*t*-Test for unpaired samples with a significance level of p = 0.05. The numbers of patients having high and medium loads were indicated in Figure 2.

### Analysis of Load Dependency on Muscular Strength

Due to observations from previous measurements and theoretical considerations, we expected that the patient’s muscular strength influences the maximum loads during the exercises, assuming that strong patients would produce high loads during isometric exercises (#5, 6, and 7). When exercising against gravity (e.g. #8, 9), however, the loads were expected to remain at the lowest possible limits, determined by the patient’s anthropometric data as segment masses and lever arms of masses and muscles.

Patients were grouped into those being physically active or passive. The ‘active’ group consisted of patients 1, 3, and 5, who frequently practiced sports like biking, hiking, or swimming. Patients 2, 4, and 6, who didn’t practice any sports, were assigned to the ‘passive’ group. The forces during exercises # 5, 6, 7, 8, and 9 were analyzed and compared between groups using a Student’s-*t*-test to test the assumptions.

## Results

### Time-load Patterns

**Figure 3:**
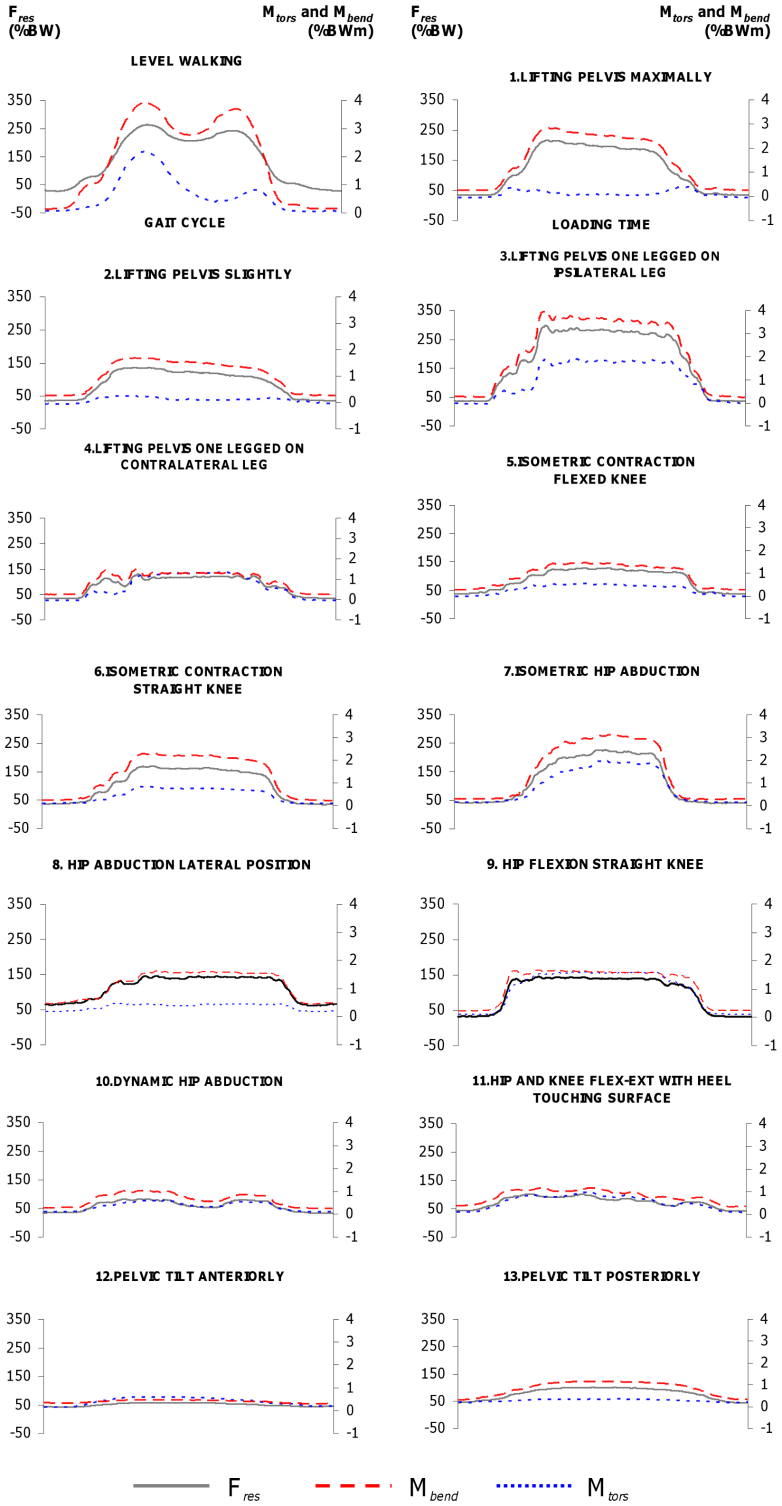
Hip joint loading during reference activities and exercises 1–13. Resultant contact force F*_res_* (black line, left axis), torque M*_tors_* around implant shaft (dotted blue line, right axis) and bending moment M*_bend_* in femoral neck (dashed red line, right axis). The x-axis indicates the loading time.

Figure 3 shows the activity-specific time-load patterns for each exercise. The pattern of level walking showed two peaks for F*_res_*, M*_tors_*, as well as M*_bend_*: the first peaks were F*_res_* = 263%BW, M*_tors_* = 2.2%BWm, and M*_bend_* = 3.9%BWm on average. The second peaks were lower with F*_res_* = 242%BW, M*_tors_* = 0.8%BWm and M*_bend_* = 3.7%BWm. The average loads during the two-legged stance were F*_res_* = 93%BW, M*_tors_* = 0.2%BWm, and M*_bend_* = 1.3%BWm.

Throughout all activities, the time-load patterns of M*_bend_* closely resembled those of F*_res_*. The same was found for M*_tors_* with the exception of exercises #1 (lifting pelvis maximally), #2 (lifting pelvis slightly), #8 (hip abduction lateral position), and #13 (tilting the pelvis posteriorly), in which the activity-specific M*_tors_* moment remained close to zero.

### Load Maxima

Figures 2A–C depict the numerically determined medians and ranges of the peak values for F*_res_*, M*_tors_* and M*_bend_*, obtained from the 36 trials (30 for exercise #4) of all subjects. Level walking at 100% (full weight bearing) and 50% (half body weight = partial weight bearing) served as individual references. The *median* 50% levels of all 3 evaluated loads *from all subjects* were with 130%BW lightly higher than the median levels during a one-legged stance (approximately 100%BW). The numbers in the upper triangles indicate the number of subjects for which an exercise caused *individual* median peak loads which were significantly higher than the *individual* median peak loads during walking (‘high loads’). The lower triangles indicate the number of patients whose loads were significantly higher than the individual 50% levels but lower than the 100% levels and therefore graded as ‘medium’ loads.

#### Resultant force F_res_

The median peak value of F*_res_* during walking, i.e. the 100% level, was 266%BW. The weight bearing exercise #3 (one-legged bridging, standing on the operated leg) was the only exercise for which the median peak force *of all subjects* exceeded 100%, i. e. the reference during walking (median 303%BW, range 225–441%BW). Although the median peaks of exercises #1, #5, #6, and #7 (weight bearing or isometric exercises) were lower than during walking, the 99th percentiles exceeded the 100% level or came close to it. Only during exercise #1 did 3 patients have high loads. In the remaining exercises, the 99^th^ percentiles were lower than the 1^st^ percentile for level walking and in none of the patients high forces were found.

#### Torsional moment M_tors_

The median peak value during walking was 2.2%BWm. Similarly to the observations for the force, the median peak torque during weight bearing exercise #3 was close to 100% (2.0%BWm, 1.0 to 3.6%BWm). In three of the subjects, high moments were found. The 99^th^ percentiles of exercises #4, #5, and #7 (weight bearing or isometric exercises) exceeded the 100% level. During exercise #4, one patient had high values of *M_tors_* and 2 patients during exercise #7. The 99^th^ percentiles of exercises #6, #9, #11, and #13 did not reach 100%, but approached it closely, with one patient having high values. For exercises #1, 2, 8, and 13, the peak values ranged from negative values of −0.7%BWm to positive 1.5%BWm, i.e., the medians were distributed around zero.

#### Bending moment M_bend_

The median peak value during walking was 3.9%BWm. As for force and torque, exercise #3 also caused the highest bending moment of all the exercises. The median was higher than 100% (4.0 BWm, 3.2 to 5.4%BWm). Three of the patients had high values of *M_bend_*. During other weight bearing and isometric exercises (#1, #2, #5, #6, and #7), the 99^th^ percentiles exceeded the reference value; 1 subject had high values. During the exercises #4, #8, #9, #10#, #11, #12, and #13, the 99^th^ percentiles remained below 100%.

### Load Dependency on Muscular Strength

From the isometric exercises, #7 revealed a statistically significant difference between the active and the passive group (#7: active 241%BW, passive 180%BW, *p*<0.01). During exercise #5 and #6, the median peak forces showed small differences (#5: active 120%BW, passive 144%BW, *p = *0.35; #6: active 177%BW, passive 171%BW, *p* = 0.69), but no trend towards higher loads in the active group. For the exercises with long lever arms, a significant difference between groups was observed when flexing the hip in supine position by raising the leg (#9: active 140%BW, passive 154%BW, *p*<0.01) but abducting the leg in lateral position did not show any notable differences (#8: active 146%BW, passive 149%BW, *p* = 0.51).

## Discussion

This study addressed the question of how demanding post-operative physiotherapeutic hip exercises are by determining the acting hip joint forces and moments with instrumented implants.

After hip surgery, physiotherapy is important to mobilize the patient and restore his function. The physiotherapist’s aim is thereby to increase muscle strength, improve joint mobility and train activities, enabling the patient to live as independently as possible. To ensure optimal initial bone ingrowth around the implant, load-dependent micromotions at the bone-stem-interface must be minimized as they may otherwise prevent implant stabilization and cause loosening. Similarly, high loads acting at fracture implants may cause non-union or pseudarthrosis. Orthopedic surgeons are confronted with the conflict between permitting unrestricted weight bearing for fast recovery and avoiding high mechanical loading that may cause complications and hinder fracture consolidation. Additionally, walking with partial weight bearing or only floor contact requires a considerable amount of muscle strength in the upper extremities and trunk, so it is hardly achievable for many elderly patients [3], [4]. These may be reasons why rehabilitation protocols vary between clinics. One study found large diversity in rehabilitation protocols [12]: out of 53 surveyed surgeons, 38 allowed full weight bearing for uncemented implants, yet 10 prescribed partial weight bearing with half body weight and 3 allowed only toe-touch weight bearing. Only 9 surgeons reported that their protocols were evidenced-based, but no detailed information was provided.

Among all exercises, the highest median peak loads were observed for the Lifting Pelvis weight bearing exercises (#1–4). When Lifting Pelvis was performed with support only by the operated leg (#3), the median peak forces and moments exceeded 100%, i.e. the values during walking, in 3 to 4 patients. In one trial, F*_res_* rose up to even 441%BW equaling 166% of walking with full weight bearing. When the pelvis was lifted only slightly (#2), the median peak of F*_res_* reached 82% and were therefore in the medium range. Some physicians disapprove Lifting Pelvis as a bed exercise in the early post-operative period, but it should be taken into account that the same activity is necessary when using a bedpan. Fleischhauer (2006) recommends exercise #4 (Lifting Pelvis standing only on the contralateral leg while the operated leg is lifted with extended knee) to be practiced directly after pelvic osteotomies [24] because it is commonly believed that a non-weight bearing joint is unloaded. In our study, this exercise caused a medium hip contact force above 50%. The torsional moment reached values close to 100% in some trials. Such load levels in a non-weight bearing joint can be explained by co-contraction of the muscles crossing the hip joint as any muscular co-contraction unavoidably increases the joint contact force.

The force-increasing effect of co-contractions can also be observed during isometric exercises. Fundamental biomechanical reasons suggest that the theoretically achievable ultimate levels depend on the intensity of the muscle contraction and therefore on the muscle strength. We did not find notable differences between active and passive patients. The assignment to the two cohorts was based on subjective observations, however, and the maximum voluntary muscle strength had not been quantified. Nevertheless, our data suggest that the contraction intensity depends on multiple factors such as the patient’s motivation and/or the instructions given by the physiotherapist rather than the maximum strength. Still, according to our observations, high intensive contractions may lead to high joint loads during isometric exercises. If fractures with uncertain stability prohibit high loads at the fracture site, the physiotherapist should therefore avoid high intensity muscle contractions by checking the contraction by palpation and controlling it by verbal instructions.

**Figure 4:**
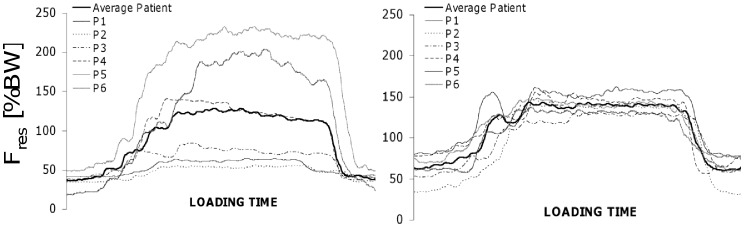
Patient- and activity-specific time courses of resultant force F*_res_*. Left: Exercise #5 = isometric contraction with flexed knees. Right: Exercise #7 = hip abduction in lateral position. Data from 6 patients. The curves of the isometric contraction reveal a broad scattering of the peak values, ranging from 56 to 232%BW. This range is due to different voluntary muscular contraction intensities and depends strongly upon the patient’s motivation and the instructions given by the physiotherapist. When abducting the straight leg in the lateral position, peak loads range only slightly between 130 and 162%BW. This is a result of biomechanical factors such as similar leg lengths, segment masses and lever arms of the gluteal musculature.

In contrast to the widely varying forces during isometric co-contractions, the loads when exercising against gravity can be predicted relatively precisely from our data (Figure 4). The individual forces during flexion or abduction of the straight leg, for example, remained in a close range between 49 and 68%BW for #8 and 50 and 69%BW for #9. The individual bending moments were also similar during flexion and abduction. The torsional moment, however, was 7-times higher during flexion than during abduction. This is due to the high anteroposterior force component F*_y_*_′_ when flexing the hip joint. During exercises #1, #2, #8, and #13 M*_tors_* was distributed around zero when the data from all subjects were averaged, which was a result of individually different signs of F*_y_*_′_ and therefore of M*_tors_*. These varying force directions may be a result of different hip joint anatomy, particularly the implant anteversion. When the pelvis was tilted anteriorly and posteriorly (#12 & 13), M*_tors_* even changed its sign within the movement in 4 patients, a factor that may increase the risk of delayed bone formation at the implant’s interface.

Dynamic exercises with an open kinetic chain (non-weight bearing conditions) and short lever arms (#10–13) caused low peak forces of approximately 38%, torque between 23% and 45% and bending moments between 13% and 26%. These are values classified as low, but even much lower loads had been expected, because the moved body parts were supported by the therapy table and had therefore not to be lifted against gravity. This again demonstrates the decisive impact of the muscles on the internal loads.

It remains unclear whether the load magnitudes during walking ( = 100%) are the critical upper loading limits. Furthermore, the primary stability varies from case to case and was not focus of this study so that statements about primary stability cannot be given. However, orthopedic surgeons should take the following into account when deciding on partial (or even toe-touch) weight bearing: unavoidable activities such as using a bed pan and even some bed exercises cause medium to high loads. If reduced weight bearing is nevertheless demanded by the surgeon, the physiotherapeutic exercises shown here to produce medium or high loads should consequently be omitted from physiotherapeutic treatment. Vice versa, the patient should be allowed to walk with full weight bearing if these exercises are thought to be tolerable. As muscle strengthening is a major aim of physiotherapeutic treatment and necessary for recovery, it should be discussed whether strengthening exercises with intensive muscle contraction shall be avoided.

This study has some limitations. We investigated only 6 subjects so that reliable and generally representative conclusions are difficult to be drawn. Additionally, the assignment to the active and passive group was only based on the sports activities reported by the patients. The muscular strength had not been quantified.

Furthermore, position changes between the single physiotherapeutic exercises could possibly lead to high loads. We did not evaluate these movements but instead collected the exercise data in a systematic manner for best averaging accuracy and intra-individual comparison. This method enabled us to note tendencies and provide unique data that have not been previously obtained. The findings of this study give important scientific information about *in vivo* loading during physiotherapeutic exercises and will support orthopedic surgeons, therapists and patients in their decision making and help to develop effective and individual rehabilitation protocols.

## Conclusions

Weight bearing activities caused the highest loads among all exercises. Movements against resistance or loads acting at long lever arms seem to be non-hazardous regarding the force magnitudes, but may cause high torsional moments. The forces during isometric contractions depend on the contraction intensity which is rather influenced by the motivation than by the maximal muscle strength. Generally, the joint contact forces are increased by muscle co-contractions, which press the joint partners against each other, an effect that is observed when exercising the contralateral limb while the ipsilateral limb is passive. When deciding between partial and full weight bearing, physicians should consider the loads relative to those observed during walking.

## References

[1] Kamel HK, Iqbal MA, Mogallapu R, Maas D, Hoffmann RG (2003) Time to Ambulation After Hip Fracture Surgery: Relation to Hospitalization Outcomes. The Journals of Gerontology Series A: Biological Sciences and Medical Sciences 58: M1042–M1045. 10.1093/gerona/58.11.m1042 14630887

[2] Oldmeadow LB, Edwards ER, Kimmel LA, Kipen E, Robertson VJ, et al (2006) No rest for the wounded: early ambulation after hip surgery accelerates recovery. ANZ Journal of Surgery 76: 607–611. 16813627 10.1111/j.1445-2197.2006.03786.x

[3] Vasarhelyi A, Baumert T, Fritsch C, Hopfenmüller W, Gradl G, et al (2006) Partial weight bearing after surgery for fractures of the lower extremity–is it achievable? Gait & Posture 23: 99–105. 16311201 10.1016/j.gaitpost.2004.12.005

[4] Jöllenbeck T (2005) Die Teilbelastung nach Knie- und Hüft-Totalendoprothesen: Unmöglichkeit ihrer Einhaltung, ihre Ursachen und Abhilfen. Z Orthop Ihre Grenzgeb 143: 124–128. 15849615 10.1055/s-2005-868436

[5] Burke DW, O’Connor DO, Zalenski EB, Jasty M, Harris WH (1991) Micromotion of cemented and uncemented femoral components. The Journal of Bone and Joint Surgery British Volume 73: 33–37. 1991771 10.1302/0301-620X.73B1.1991771

[6] Piliar R (1986) Observation on the Effect of Movement on Bone Ingrowth into Porous-Surfaced Implants. Clin Orthop Relat Res 208: 108–113. 3720113

[7] Bragdon CR, Burke D, Lowenstein JD, Connor DOO, Ramamurti B, et al (1996) Differences in Stiffness Between a Cementless and Cancellous Bone into Varying Amounts of of the Interface Porous Implant vivo in Dogs Due Implant Motion. Clin Orthop 11: 945–951. 10.1016/s0883-5403(96)80136-7 8986573

[8] Claes L, Fiedler S, Ohnmacht M, Duda GN (2000) Initial stability of fully and partially cemented femoral stems. Clinical Biomechanics (Bristol, Avon) 15: 750–755. 10.1016/s0268-0033(00)00044-9 11050357

[9] Bieger R, Ignatius A, Decking R, Claes L, Reichel H, et al (2011) Primary stability and strain distribution of cementless hip stems as a function of implant design. Clinical Biomechanics 27: 158–164. 21889243 10.1016/j.clinbiomech.2011.08.004

[10] Heller M, Kassi J-P, Perka C, Duda G (2005) Cementless stem fixation and primary stability under physiological-like loads in vitro. Biomed Tech (Berl) 50: 394–399. 16429942 10.1515/BMT.2005.054

[11] Zwartelé RE, Witjes S, Doets HC, Stijnen T, Pöll RG (2012) Cementless total hip arthroplasty in rheumatoid arthritis: a systematic review of the literature. Archives of Orthopaedic and Trauma Surgery 132: 535–546. 22113434 10.1007/s00402-011-1432-0 PMC3306565

[12] Hol A, Van Grinsven S, Rijnberg Wj, Susante J, Van Loon C (2006) Varatie in nabehandeling can de primaire totaleheupprothese via de posterolaterale benadering. Nederlands Tijschrift voor Orthopaedie 13e: 105–113.

[13] Thomas S, Mackintosh S, Halbert J (2011) Determining current physical management of hip fractrue in acute care hospital and physical therapists’ rationale for this management. Phys Ther 91: 1490–1502. 21817011 10.2522/ptj.20100310

[14] Yang H, Zhou F, Tian Y, Ji H, Zhang Z (2011) Analysis of the failure reason of internal fixation in periptrochanteric fractures. Journal of Peking Inversity (Helath Sciences) 43: 699–702. 22008679

[15] Lorich DG, Geller DS, Nielson JH (2004) Osteoporotic Pertrochanteric Hip Fractures. Management and Current Controversies. The Journal of Bone and Joint Surgery 86-A: 398–410. 15116633

[16] Van Vugt AB (2007) Femoral neck non-unions: How do I do it? Injury, Int J Care Injured 38S: 51–54.

[17] Knobe M, Münker R, Sellei R, Schmidt-Rohlfing B, Erli H, et al (2009) Die instabile pertrochantäre Femurfraktur. Komplikationen, Fraktursinterung und Funktion nach extra- und intramedullärer Versorgung (PCCP™, DHS und PFN). Zeitschrift für Orthopädie und Unfallchirurgie 147: 306–313. 19551581 10.1055/s-0029-1185349

[18] Bergmann G, Deuretzbacher G, Heller M, Graichen F, Rohlmann A, et al (2001) Hip contact forces and gait patterns from routine activities. Journal of Biomechanics 34: 859–871. 11410170 10.1016/s0021-9290(01)00040-9

[19] Bergmann G, Graichen F, Rohlmann A (1995) Is staircase walking a risk for the fixation of hip implants? Journal of Biomechanics 28: 535–553. 7775490 10.1016/0021-9290(94)00105-d

[20] Bergmann G, Graichen F, Rohlmann A (2004) Hip joint contact forces during stumbling. Langenbeck’s Archives of Surgery/Deutsche Gesellschaft für Chirurgie 389: 53–59. 10.1007/s00423-003-0434-y 14625775

[21] Bergmann G, Rohlmann A, Graichen F (1989) In vivo Messung der Hüftgelenkbelastung 1. Teil: Krankengymnastik. Z Orthop 127: 672–679. 2618148 10.1055/s-2008-1040311

[22] Damm P, Graichen F, Rohlmann A, Bender A, Bergmann G (2010) Total hip joint prosthesis for in vivo measurement of forces and moments. Medical Engineering & Physics 32: 95–100. 19889565 10.1016/j.medengphy.2009.10.003

[23] Bender A, Bergmann G (2011) Determination of typical patterns from strongly varying signals. Computer Methods in Biomechanics and Biomedical Engineering: 37–41. 10.1080/10255842.2011.560841 21722048

[24] Fleischhauer M (2006) Leitfaden Physiotherapie in der Orthopädie und Traumatologie. 2. ed. Fleischhauer M, Heimann D, Hinkelmann U, editors Urban & Fischer München.

